# A proposed model to evaluate how changes in body condition score and the fatty acid profile of a supplement affect physiology and metabolic responses of nonlactating females

**DOI:** 10.3168/jdsc.2022-0349

**Published:** 2023-07-13

**Authors:** Carlos Eduardo Dias e Silva, Victor Miranda, Miguel Miranda, Júlia G. Silva, Isabella R.T. Souza, Samir Burato, Osvaldo A. de Sousa, Ronaldo L.A. Cerri, Fábio S. Lima, Bruno I. Cappellozza, José Luiz M. Vasconcelos

**Affiliations:** a School of Veterinary Medicine and Animal Science, São Paulo State University (UNESP), Botucatu 18168-000, Brazil; b Nutricorp, Araras 13609-899, Brazil; c Faculty of Land and Food Systems, University of British Columbia, Vancouver, BC V6T 1Z4, Canada; d Department of Population Health and Reproduction, School of Veterinary Medicine, University of California, Davis, CA 95616

## Abstract

Two experiments were designed to evaluate the effects of altering body condition score (BCS) and the profile of a fatty acid (FA) supplement on the metabolism of *Bos indicus* Nellore females. In experiment 1, 16 and 24 *B. indicus* heifers and nonlactating cows, respectively, were assigned to (1) maintenance diet (MNT-MNT; n = 10), (2) maintenance diet and BCS loss (MNT-LSS; n = 10), (3) maintenance diet supplemented with calcium salts of soybean oil for 30 d and BCS loss for 40 d (MNT+CFA-LSS; n = 10), and (4) maintenance diet for 30 d and BCS loss for 40 d with a diet containing calcium salts of soybean oil (MNT-LSS+CFA; n = 10). Following the BCS loss period, MNT-LSS, MNT+CFA-LSS, and MNT-LSS+CFA were fed a diet to promote the gain of BCS. In experiment 2, 40 *Bos indicus* nulliparous heifers were assigned to (1) maintenance diet (MNT-MNT; n = 10), (2) BCS loss followed by a BCS gain (LSS-REM; n = 10), (3) BCS loss followed by a BCS gain diet with CFA of palm oil (LSS-REM+PLM; n = 10), and (4) BCS loss followed by a BCS gain diet with CFA of soybean oil (LSS-REM+SOY; n = 10). Blood samples were obtained for serum haptoglobin and fecal samples for pH (experiment 2 only). In experiment 1, a treatment × day interaction was observed for BCS during the 60-d BCS loss and gain period. Animals assigned to MNT-MNT had a greater BCS than the other treatment groups on d 40 and 60 of the experiment, but no other differences were observed. Moreover, a treatment × day interaction was observed for serum haptoglobin, as on d 60, MNT-LSS had a greater mean serum haptoglobin concentration. In experiment 2, a treatment × day interaction was also observed for BCS. From d −4 to 0, LSS-REM and LSS-REM+SOY had a reduced BCS versus MNT-MNT, but also lower for LSS-REM versus MNT-MNT on d 1, and LSS-REM+PLM versus MNT-MNT on d −1 and 0. For serum haptoglobin, no treatment or treatment × day interaction was observed. A treatment × day interaction was observed for fecal pH. From d −10 to 0, MNT often had a lower fecal pH, but during realimentation, LSS-REM heifers had a reduced fecal pH on d 1, 4, and 10. In summary, we failed to demonstrate an increase in serum haptoglobin due to a BCS loss. Still, supplementation with calcium salts of FA alleviated the increase in haptoglobin and maintained fecal pH at more stable values during realimentation, regardless of the FA profile of the supplement.

## Highlights

• BCS fluctuation caused by changes in DMI may lead to health, productive, and reproductive losses to the herd.
• Due to their nutraceutical properties, calcium salts of polyunsaturated fatty acids might support the health of the herd during periods of BCS changes.
• Feed restriction effectively reduced BCS, but no changes were observed on haptoglobin.
• Supplementation of CFA alleviated the increase in haptoglobin and maintained fecal pH at higher levels following realimentation.

## Graphical Abstract

**Figure:**
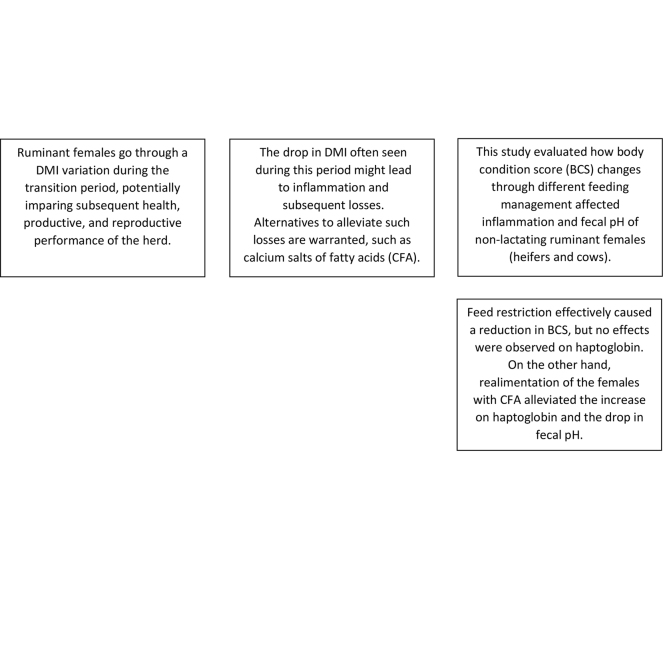
**Summary:** The transition period of a ruminant female is characterized by changes in dry matter intake (DMI) that ultimately affects body condition score (BCS) and inflammatory markers. To date, few studies have evaluated how BCS changes (loss and gain) over time affect inflammatory markers. Therefore, we evaluated how BCS changes and feeding of calcium salts of fatty acids (CFA) would affect haptoglobin and fecal pH. Overall, providing a greater amount of feed led to greater haptoglobin concentrations, whereas feeding CFA alleviated this response and maintained fecal pH at higher levels. In summary, feeding management may affect performance and inflammation of the herd.

Body condition score loss is often observed in transition dairy cows, mainly caused by a reduction in DMI (Janovick and Drackley, 2010; Elolimy et al., 2022). This reduction in prepartum DMI and, consequently, alteration in prepartum BCS has been linked to impaired immunity (Hammon et al., 2006); increased incidence of health disorders (Huzzey et al., 2007); lower milk yield, quality, and composition; and poor reproductive performance (Dennis et al., 2022). Recently, Dennis et al. (2022) demonstrated that dairy cows that lost BCS between dry-off and calving were more likely to be diagnosed with postpartum diseases and be treated with antibiotics. It is noteworthy that most research efforts have focused on understanding the metabolic and inflammatory pathways of heifers and cows close to calving and immediately postcalving, whereas postcalving responses might arise during precalving. As an example, previous research underscored that DMI restriction stimulates an exacerbated inflammatory response (Marques et al., 2019). Therefore, it is reasonable to surmise that heifers and cows might be susceptible to exacerbated inflammatory states close to calving, with haptoglobin concentrations often increasing between calving and 2 wk after calving (Edelhoff et al., 2020).

Moreover, considering the well-established impacts of stressors such as dietary restrictions (Horst et al., 2021) on inflammation, an increase on the severity of inflammatory state might be worsened following dietary alterations that increase the amount of concentrate in the diet. One technology to alleviate this nutrient-induced inflammation while maintaining the energy density of the diet is to offer fatty acids (**FA**; NRC, 2001) that, depending on the source, also modulate the immune response (Cappellozza et al., 2012). Based on this rationale, we hypothesized that BCS alterations caused by DMI restriction would increase haptoglobin and that DMI resumption would lead to a similar response in haptoglobin and decrease fecal pH in *Bos indicus* females. Hence, 2 experiments were conducted to evaluate (1) how BCS loss and gain modulates inflammation, (2) whether calcium salts of fatty acid (**CFA**) supplementation, (3) CFA profile, and (4) timing of CFA supplementation could be used as potential nutritional alternatives to modulate these inflammatory responses in *Bos indicus* livestock females.

Both experiments were conducted at a commercial farm (Sítio Santo Antônio, São Manuel, SP, Brazil) from October 2019 to December 2020. All animals were cared for in accordance with the experimental protocols reviewed and approved by the Animal Use and Ethics Committee of the São Paulo State University–School of Veterinary Medicine and Animal Science (0171/2019).

In experiment 1, 16 and 24 *Bos indicus* nulliparous Nellore heifers and nonlactating Nellore cows, respectively, were ranked by initial BW (heifers 360 ± 62.8 kg and cows 416 ± 50.7 kg) and BCS (heifers 3.5 ± 0.32 and cows 2.8 ± 0.31; Wagner et al., 1988) were allocated in individual pens (10 × 5 m) and assigned to (n = 4 heifers and 6 cows per group): (1) maintenance diet throughout the 90 d trial (**MNT-MNT**), (2) maintenance diet for 30 d and then BCS loss for 40 d (**MNT-LSS**), (3) maintenance diet for 30 d with the addition of calcium salts of soybean oil and then BCS loss for 40 d (**MNT+CFA-LSS**), and (4) maintenance diet for 30 d and then BCS loss for 40 d in a diet with the addition of calcium salts of soybean oil (**MNT-LSS+CFA**; Nutri Gordura, Nutricorp, Araras, SP, Brazil). The goal of having the 30-d maintenance period was dual, as (1) to bring all animals, within each treatment, into a similar BCS on d 0 of the study (approximately 3.50) and (2) to supplement the FA for an adequate period to provide the potential beneficial effects (Araujo et al., 2010; Cooke et al., 2011; Cappellozza et al., 2012). The amount of BCS loss over the 40 d was designed to be in the order of 0.75 points, achieved by restricting the feed offered to the animals daily. Following the BCS loss period (40 d), MNT-LSS, MNT+CFA-LSS, and MNT-LSS+CFA were fed a diet to promote gain of BCS (0.50 points) for another 20 d, whereas MNT-MNT animals were fed the same diet. During the initial 30 d, MNT and CFA animals received an isonitrogenous and isocaloric diet, with the difference being the inclusion or not of CFA (NRC, 2001). Similarly, during the LSS period, dietary intakes were isonitrogenous and isocaloric, and the only difference was CFA inclusion (2% of diet DM). The feedstuffs used included corn silage, whole corn grain, soybean meal, and mineral-vitamin mix, whereas nutrient intake was also recorded during the maintenance, BCS loss, and realimentation phases.

Individual BW and BCS measurements were performed on d −30, −14, 0, 20, 40, and 60 of the study. Additionally, from d 0 to 60, blood samples were collected into commercial blood collection tubes (Vacutainer, 10 mL; Becton Dickinson) containing no additive for determination of serum haptoglobin (Cooke and Arthington, 2013). Blood samples were placed immediately on ice after collection, centrifuged (2,500 × *g* for 30 min; 4°C) for serum harvest, and stored at −20°C on the same day of collection.

In experiment 2, 40 *Bos indicus* nulliparous Nellore heifers were ranked by initial BW (433 ± 23.7 kg) and BCS (4.2 ± 0.41; Wagner et al., 1988), allocated in individual pens (10 × 5 m), and assigned to (1) maintenance diet throughout the 28 d trial (**MNT-MNT**; n = 10), (2) BCS loss followed by a BCS gain period (**LSS-REM**; n = 10), (3) BCS loss followed by a BCS gain period with the addition CFA of palm oil into the diet (**LSS-REM+PLM**; Nutri Gordura Lac, Nutricorp; n = 10), and (4) BCS loss followed by a BCS gain period with the addition CFA of soybean oil into the diet (**LSS-REM+SOY**; Nutri Gordura, Nutricorp; n = 10). The amount of BCS loss in the 14 d was designed to be 0.50 points, achieved by restricting the amount of feed offered to the animals daily. Following the BCS loss period, LSS-REM, LSS-REM+PLM, and LSS-REM+SOY animals were fed a diet to promote gain of BCS for another 14 d (0.50 points), while MNT animals were fed the same diet. Throughout the experiment, dietary intakes of LSS-REM, LSS-REM+PLM, and LSS-REM+SOY were isonitrogenous and isocaloric, the only difference being CFA inclusion (2% of diet DM). The feedstuffs utilized included corn silage, whole corn grain, soybean meal, and mineral-vitamin mix. Nutrient intake was also recorded during the maintenance, BCS loss (d 0 to 20 and 20 to 40), and realimentation (d 40 to 60) phases.

Individual BW and BCS measurements were performed on d −14, −10, −7, −4, −1, 0, 1, 4, 7, 10, and 14 of the study. Concurrently with these assessments, blood samples were collected into commercial blood collection tubes (Vacutainer, 10 mL; Becton Dickinson) containing no additive for the determination of serum haptoglobin (Cooke and Arthington, 2013). Blood samples were placed immediately on ice after collection, centrifuged (2,500 × *g* for 30 min; 4°C) for serum harvest, and stored at −20°C on the same day of collection. Moreover, fecal samples (approximately 50 g; Onyx digital scale; Clink) were manually collected directly from the rectum of each animal for pH determination using a digital portable pH reader (AET15 pH Tester; Fisher Scientific).

In both experiments, the FA profile of the individual feedstuffs were analyzed (Universidade de Campinas, Campinas, SP, Brazil), the exception being the CFA that have been analyzed by the manufacturer and the results used for FA intake of the animals. Moreover, it is noteworthy that *Bos indicus* Nellore females were used in both experiments to obtain animals that have not been (for heifers) or were not pregnant (for cows) and to mitigate any risk of a previous disease event that could have a carryover effect in the responses of the current experiments.

All data were analyzed using the animal as the experimental unit, using the MIXED procedure of SAS (version 9.4; SAS Institute Inc.), and Satterthwaite approximation to determine the denominator degrees of freedom for tests of fixed effects. In experiment 1, the model statement contained the effects of treatment, parity (cows or heifers), day, and all the resultant interactions. The random statement included pen. The specified term for repeated statements was day (for BCS, BW, and haptoglobin) or period (for nutrient intake), with pen (treatment) as the subject, and the covariance structure used was autoregressive, which provided the smallest Akaike information criterion and hence the best fit for all variables analyzed herein. In experiment 2, the model statement contained the effects of treatment, day, and the resultant interaction. The random statement included pen. The specified term for repeated statements was day, with pen (treatment) as the subject, and the covariance structure used was autoregressive. Samples (blood and feces) collected on d −14 were used as covariates for subsequent analysis. Significance was set at *P* ≤ 0.05, and tendencies were determined if *P* > 0.05 and ≤0.10. Repeated measures are reported according to the main treatment effect if no higher-order interactions were detected.

During the 30-d maintenance period, no treatment effects were observed for BCS (*P* = 0.66) and BW (*P* = 0.56), but parity was significant for both variables (*P* < 0.0001; data not shown). Conversely, and as expected, a treatment × day interaction was observed (*P* < 0.0001) for BCS during the remaining 60-d period of the trial (BCS loss and gain; Figure 1A). Animals assigned to MNT-MNT had a greater BCS than the other treatment groups on d 40 and 60 of the experiment (*P* ≤ 0.02), whereas no differences were observed between MNT-LSS, MNT+CFA-LSS, MNT-LSS+CFA (*P* ≥ 0.73). Nonetheless, it is important to mention that the BCS loss achieved during this period was equal to 0.60, whereas the goal was 0.75. Our results demonstrate that the experimental design adopted and proposed herein was effective in (1) maintaining BCS during the initial period, (2) causing the BCS loss in the subsequent 40-d period, and (3) isolating the potential effects of CFA supplementation, if any, on the FA profile of the supplement and not on energy intake per se. In support of the latter statement, a treatment by day interaction was observed for serum haptoglobin concentrations (*P* = 0.02; Figure 1B). Significant differences were not observed on d 0 and 20 of the trial (*P* ≥ 0.23). Still, on d 40, serum haptoglobin tended to be greater in MNT-LSS versus MNT-MNT (*P* = 0.07), and on d 60, corresponding to 20 d of realimentation and BCS gain, MNT-LSS had a greater mean serum haptoglobin concentration versus all other treatments (*P* ≤ 0.01; Figure 1B).

**Figure 1:**
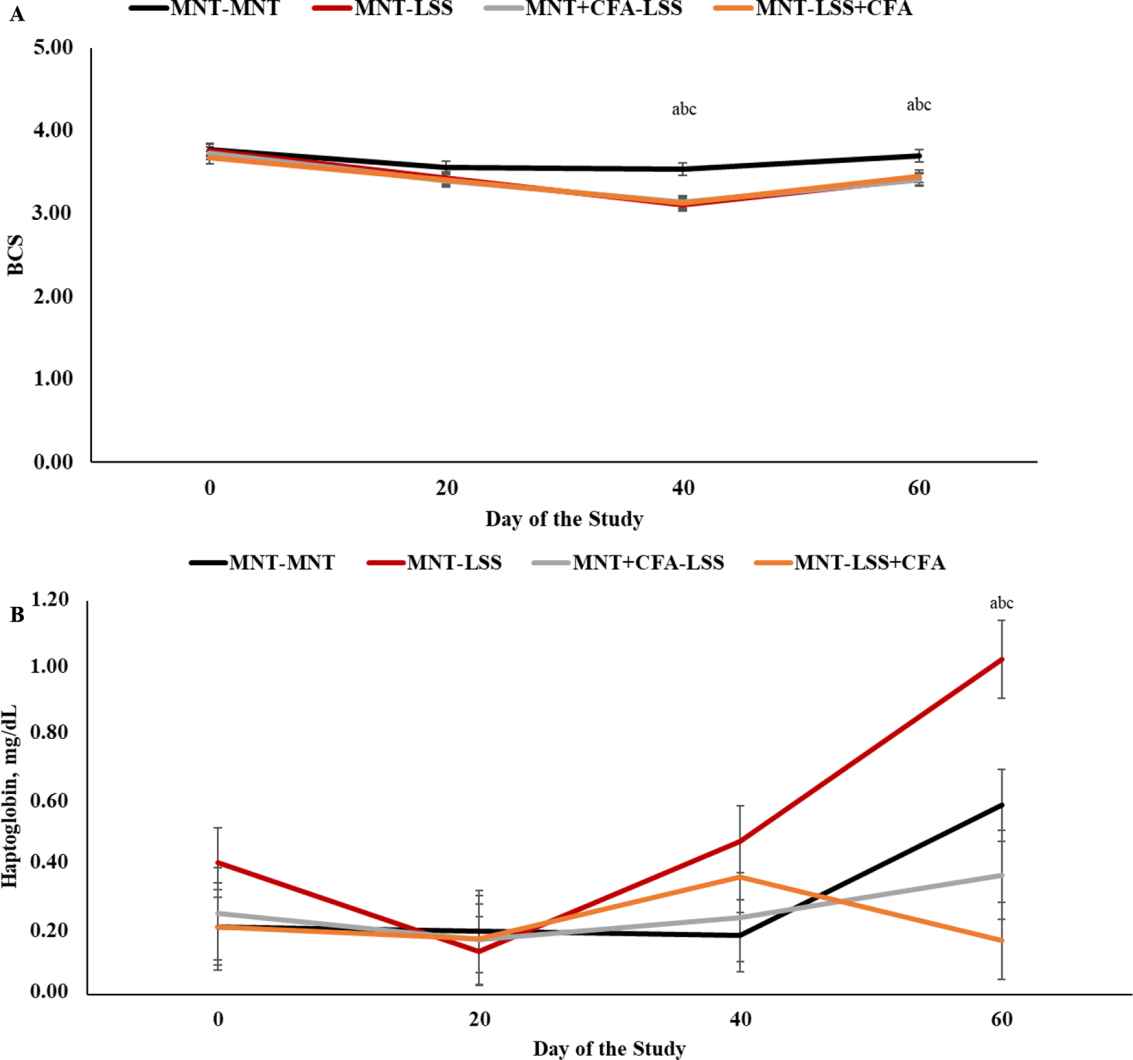
Body condition score (A) and serum haptoglobin concentration (B) of heifers and cows assigned to (1) maintenance diet throughout the 90-d trial (MNT-MNT), (2) maintenance diet for 30 d and then BCS loss for 40 d (MNT-LSS), (3) maintenance diet for 30 d with the addition of calcium salts of soybean oil and then BCS loss for 40 d (MNT+CFA-LSS), and (4) maintenance diet for 30 d and then BCS loss for 40 d in a diet with the addition of calcium salts of soybean oil (MNT-LSS+CFA; Nutri Gordura, Nutricorp, Araras, SP, Brazil). A treatment × day interaction was observed for BCS (*P* < 0.0001; SEM = 0.074) and serum haptoglobin (*P* = 0.02; SEM = 0.119). Panel A: a = MNT-MNT versus MNT-LSS (*P* ≤ 0.01); b = MNT-MNT versus MNT+CFA-LSS (*P* ≤ 0.01); c = MNT-MNT versus MNT-LSS+CFA (*P* ≤ 0.02). Panel B: a = MNT-LSS versus MNT-MNT (*P* < 0.01); b = MNT-LSS versus MNT+CFA-LSS (*P* ≤ 0.01); c = MNT-LSS versus MNT-LSS+CFA (*P* ≤ 0.01).

In dairy cattle, cows that lost BCS during precalving had a reduced health function, reproductive performance, and fat-corrected milk production versus cows maintaining and gaining BCS during the same period (Dennis et al., 2022). Dry matter intake is known to be reduced close to calving, indicating that BCS losses can be mediated by the reduced DMI, leading to inflammation during this period (Marques et al., 2019). More specifically, the body tissue mobilized during feed deprivation may be recognized by the immune system, leading to inflammation (Abbas and Lichtman, 2007) and yielding an even more significant reduction in DMI. Additional factors might be involved in this reduced DMI and, therefore, BCS loss, such as the macrophages that inhabit body fat and produce leptin (Schneiderman et al., 2012), a hormone known to regulate DMI in ruminants (Rodrigues et al., 2015). The lack of treatment effects on serum haptoglobin concentrations during the period of DMI restriction and, therefore, BCS loss does not agree with our hypothesis, as others have demonstrated that DMI restriction leads to an inflammatory response in ruminants (Marques et al., 2019). So, it can be speculated that in the present experiment, the feed restriction and BCS variation were not at the magnitude needed to trigger an inflammatory response and an increased serum haptoglobin, as losses of ≥0.75 promote the greatest number of negative results in dairy cows (Dennis et al., 2022) or the fact that a disease might trigger inflammation during the precalving period, leading to an increase in haptoglobin immediately postcalving (Edelhoff et al., 2020). Nonetheless, the fact that during realimentation, MNT-LSS had a greater mean haptoglobin at the end of the study is a novel and exciting finding of the present study.

As CFA replaced corn, a reduced starch intake was expected in MNT-CFA versus MNT-LSS. As reported in Table 1, MNT-CFA had a reduced starch intake versus MNT-LSS and CFA-LSS during the realimentation phase (*P* = 0.03), without differences between MNT-LSS and CFA-LSS (*P* = 0.88). Overall, MNT animals had the greatest nutrient intake from d 0 to 40 (BCS loss phase) but the lowest nutrient intake in the last 20 d of the experimental period (Table 1). The realimentation period consisted of an abrupt change in the amount of feed being offered to the animals, which can predispose animals to ruminal disorders (Owens et al., 1998), leading to inflammation and an increase in the hepatic gene expression and circulation of acute-phase proteins, such as haptoglobin, in dairy cows (Gozho et al., 2006; Plaizier et al., 2018; Lai et al., 2022; Meng et al., 2022). On the other hand, CFA inclusion either during maintenance (MNT+CFA) or BCS loss (MNT-LSS+CFA) phases was able to alleviate the increase in serum haptoglobin, likely due to the presence of essential, linoleic, and linolenic FA, which have been shown to modulate the immune response in ruminants (Cooke et al., 2011; Cappellozza et al., 2012). Based on Table 1, one might argue that no differences were observed in the intake of linoleic and linolenic acids among the LSS groups. Still, it is essential to mention that blood concentrations of these FA have not been evaluated. Intake of FA was estimated based on DMI, not considering possible rumen metabolism of these FA by specific bacteria (Jenkins et al., 2008).

**Table 1:** Nutrient intake during the BCS loss and gain periods (d 0–40 and 40–60, respectively) in heifers and cows assigned to experiment 1^1^

Item	Treatment				SEM	*P*-value^2^	
	MNT-MNT	MNT-LSS	MNT+CFA-LSS	MNT-LSS+CFA		T	T × P
DM, kg/d					0.257	<0.0001	<0.0001
d 0–20	5.69^a^	2.81^b^	2.83^b^	2.79^b^			
d 20–40	6.66^a^	1.75^b^	1.35^b^	1.30^b^			
d 40–60	6.66^b^	10.53^a^	10.62^a^	10.12^a^			
CP, kg/d					0.029	0.35	<0.0001
d 0–20	0.54^a^	0.30^b^	0.30^b^	0.29^b^			
d 20–40	0.62^a^	0.20^b^	0.16^b^	0.15^b^			
d 40–60	0.62^b^	1.27^a^	1.28^a^	1.18^a^			
Ether extract, kg/d					0.010	<0.0001	<0.0001
d 0–20	0.20^a^	0.10^b^	0.10^b^	0.15^b^			
d 20–40	0.23^a^	0.07^b^	0.05^b^	0.07^b^			
d 40–60	0.23^b^	0.40^a^	0.41^a^	0.57^a^			
NDF, kg/d					0.097	<0.0001	<0.0001
d 0–20	2.28^a^	1.07^b^	1.08^b^	1.05^b^			
d 20–40	2.70^a^	0.65^b^	0.49^b^	0.47^b^			
d 40–60	2.70^b^	3.71^a^	3.74^a^	3.55^a^			
Starch, kg/d					0.087	<0.001	<0.0001
d 0–20	1.90^a^	0.95^b^	0.96^b^	0.90^b^			
d 20–40	2.21^a^	0.60^b^	0.46^b^	0.43^b^			
d 40–60	2.21^b^	3.66^a^	3.69^a^	3.38^a^			
Total digestible nutrients, kg/d					0.181	<0.001	<0.0001
d 0–20	3.92^a^	1.96^b^	1.97^b^	2.02^b^			
d 20–40	4.57^a^	1.23^b^	0.95^b^	0.96^b^			
d 40–60	4.57^b^	7.48^a^	7.54^a^	7.45^a^			
Linoleic acid, mg/d					12.4	<0.0001	<0.0001
d 0–20	275^a^	136^b^	137^b^	134^b^			
d 20–40	322^a^	65^b^	65^b^	63^b^			
d 40–60	322^b^	510^a^	514^a^	487^a^			
Linolenic acid, mg/d					1.8	<0.0001	<0.0001
d 0–20	42^a^	20^b^	20^b^	20^b^			
d 20–40	49^a^	9^b^	9^b^	9^b^			
d 40–60	49^b^	71^a^	72^a^	69^a^			

a,b Different superscript letters within a row denote differences at the *P* < 0.05 level.

1 MNT-MNT = maintenance diet; MNT-LSS = maintenance diet followed by a diet to cause a loss in BCS; MNT+CFA-LSS = maintenance diet with the addition of calcium salts of soybean oil followed by a diet to cause a loss in BCS; MNT-LSS+CFA = maintenance diet followed by a diet to cause a loss in BCS with the addition of calcium salts of soybean oil.

2 T = treatment effect; T × P = treatment × period.

In experiment 2, a treatment × day interaction was also observed for BCS (*P* = 0.05; Figure 2A). From d −4 to 0, LSS-REM and LSS-REM+SOY had a reduced BCS versus MNT-MNT (*P* < 0.05), but also lower for LSS-REM versus MNT-MNT on d 1 (*P* = 0.04). Moreover, BCS was reduced for LSS-REM+PLM versus MNT-MNT on d −1 and 0 of the trial (*P* < 0.01), but no differences were observed among the LSS groups (*P* ≥ 0.47; Figure 2A). For serum haptoglobin, data obtained on d −14 did differ among treatments (*P* = 0.02), but were not significant covariates (*P* = 0.57). No treatment (*P* = 0.33) or treatment × day interaction (*P* = 0.84) was observed for serum haptoglobin (1.55, 1.58, 1.45, and 1.96 mg/dL for MNT-MNT, LSS-REM, LSS-REM+PLM, and LSS-REM+SOY, respectively; SEM = 0.214). On the other hand, a treatment × day interaction was observed (*P* < 0.0001; Figure 2B) for fecal pH. From d −10 to 0, MNT often had a lower fecal pH versus LSS (*P* ≤ 0.05), but during the realimentation period, LSS-REM heifers had a reduced fecal pH versus all other groups on d 1, 4, and 10 (*P* ≤ 0.03; Figure 2B). Following these results, the variation in fecal pH between d 0 and 14 was also analyzed, considering d 0 values as 100% and determining the effects of re-alimentation on the proportional change of fecal pH. A treatment effect was observed (*P* < 0.0001), so that MNT-MNT varied positively, followed by CFA supplementation, and LSS only had the greatest negative variation, elucidating the results presented in Figure 2B (4.7, −4.0, −2.7, and −9.7%, respectively; SEM = 1.44). Corroborating the results reported in experiment 1, the DMI restriction management effectively reduced BCS of LSS, achieving approximately a 0.36-point loss during the 14-d period (goal = 0.50), which resulted in no differences in serum haptoglobin during the same period and also during the realimentation period. Nonetheless, the inclusion of CFA, regardless of type, was able to maintain fecal pH closer to the neutral level approximately 24 h postfeeding versus LSS-REM during the realimentation period, mainly due to a reduced starch intake in CFA supplements (data not shown). Additionally, it is likely that the LSS-REM heifers experienced either a rumen or hindgut acidosis. Still, additional research is warranted to understand if fecal pH drops from either side or both (Neubauer et al., 2020). Fecal pH has been used as a marker of the pH status in the small intestine (Ferreira et al., 1980), depending on the diet (Erfle et al., 1982; Schubert et al., 2022) and postfeeding time (Palladino et al., 2022). To the best of our knowledge, this is the first experiment reporting a daily variation in fecal pH for ruminants not challenged with an acidosis model (Sulzberger et al., 2016), as well as the benefits of feeding CFA in replacement of corn.

**Figure 2:**
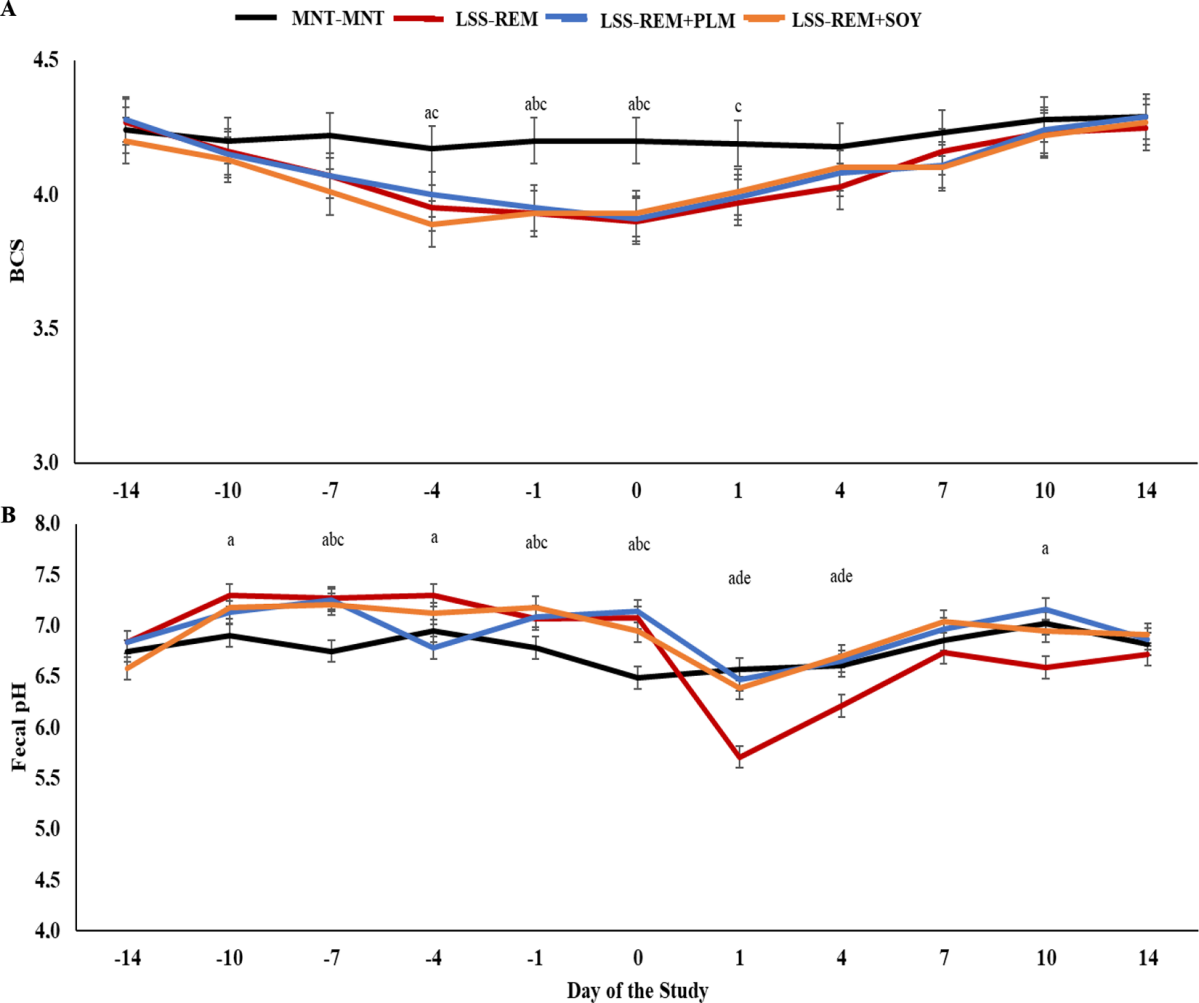
Body condition score (A) and fecal pH (B) of heifers assigned to (1) maintenance diet throughout the 28-d trial (MNT-MNT; n = 10), (2) BCS loss followed by a BCS gain period (LSS-REM; n = 10), (3) BCS loss followed by a BCS gain period with the addition CFA of palm oil into the diet (LSS-REM+PLM; Nutri Gordura Lac, Nutricorp; n = 10), and (4) BCS loss followed by a BCS gain period with the addition CFA of soybean oil into the diet (LSS-REM+SOY; Nutri Gordura, Nutricorp; n = 10). A treatment × day interaction was observed for both variables (*P* ≤ 0.05). Panel A: a = MNT-MNT versus LSS-REM (*P* < 0.05); b = MNT-MNT versus LSS-REM+PLM (*P* ≤ 0.01); c = MNT-MNT versus LSS-REM+SOY (*P* ≤ 0.03). Panel B: a = MNT-MNT versus LSS-REM (*P* ≤ 0.05); b = MNT-MNT versus LSS-REM+PLM (*P* ≤ 0.05); c = MNT-MNT versus LSS-REM+SOY (*P* ≤ 0.05); d = LSS-REM versus LSS-REM+PLM (*P* ≤ 0.04); e = LSS-REM versus LSS-REM+SOY (*P* ≤ 0.03).

In summary, the model adopted herein was adequate in yielding a reduction in BCS loss but, unfortunately, not at the magnitude to affect serum haptoglobin concentrations. Nonetheless, supplementation with calcium salts of fatty acids alleviated the increase on haptoglobin and maintained fecal pH at more stable values when the realimentation management was implemented. Caution should be taken when extrapolating our results to a dairy production setting, as beef females were used herein in an attempt to validate our initial hypothesis. Additional research is warranted to validate and evaluate the effects of DMI restriction, if any, on the health and inflammation of transition dairy cows.
